# Sex- and age-specific associations between metabolic phenotypes and brachial-ankle pulse wave velocity in adults: a cross-sectional study

**DOI:** 10.3389/fmed.2026.1915757

**Published:** 2026-09-25

**Authors:** Yi-Chih Chang, Jo-Hsuan Chen, Fang-I Liao, Wen-Cheng Li

**Affiliations:** 1 Department of Cardiology, Xiamen Chang-Gung Hospital, Hua Qiao University, Xiamen, China; 2 Department of Family Medicine, New Taipei City Municipal Tucheng Hospital, New Taipei City, Taiwan; 3 Department of Medical Education, Chang Gung Memorial Hospital at Linkou, Taoyuan, Taiwan; 4 Department of Family Medicine, Chang-Gung Memorial Hospital at Linkou, Taoyuan, Taiwan; 5 College of Medicine, Chang Gung University, Taoyuan, Taiwan

**Keywords:** arterial stiffness, Asian population, insulin resistance, metabolically healthy obesity, metabolically obese normal weight, pulse wave velocity, sex differences

## Abstract

**Objective:**

To examine age- and sex-stratified associations between metabolic phenotypes and brachial-ankle pulse wave velocity (baPWV) in adults undergoing routine health examinations.

**Methods:**

This cross-sectional study included 9,981 Chinese adults classified as metabolically healthy normal weight (MHNW), metabolically healthy obesity (MHO), metabolically obese normal weight (MONW), or metabolically unhealthy obesity (MUO) according to body mass index, homeostasis model assessment of insulin resistance, and metabolic syndrome status. Elevated baPWV was defined as mean bilateral baPWV >1700cm/s. Logistic regression analyses were stratified by sex and age (<50 and ≥50 years) and adjusted for age, smoking, and alcohol consumption. False discovery rate correction, phenotype-by-sex interaction testing, Firth logistic regression, and continuous-baPWV sensitivity analyses were performed.

**Results:**

Elevated baPWV was present in 7.5% of participants. Compared with MHNW, MONW (adjusted OR range, 2.58–7.68) and MUO (adjusted OR range, 2.34–6.60) were associated with higher odds of elevated baPWV in all four sex-age strata, with associations remaining significant after false discovery rate correction. MHO was not significantly associated with elevated baPWV. The adjusted phenotype-by-sex interaction was not significant (*P* = 0.495). Firth and continuous-baPWV sensitivity analyses yielded concordant findings, although estimates in women aged <50 years were imprecise because of sparse events.

**Conclusion:**

MONW and MUO, but not MHO, were consistently associated with less favorable baPWV profiles across sex and age strata. Assessing metabolic health alongside body mass index may provide additional information for vascular risk characterization. Longitudinal multicenter studies are needed to confirm temporal and clinical significance.

## 1 Introduction

Arterial stiffness is a hallmark of vascular aging that reflects progressive structural and functional remodeling of the arterial wall. With advancing age and cumulative exposure to cardiometabolic stressors, fragmentation and loss of elastin, increased collagen deposition and cross-linking, vascular calcification, vascular smooth muscle cell stiffening, oxidative stress, low-grade inflammation, and endothelial dysfunction reduce arterial compliance and accelerate pressure-wave transmission (1). Increased stiffness of the large elastic arteries is accompanied by higher systolic and pulse pressures, greater left ventricular afterload, and enhanced transmission of pulsatile energy to low-resistance microvascular beds, including the brain and kidneys. However, the epidemiology of arterial stiffness cannot be summarized by a single global prevalence because studies use different vascular segments, pulse wave velocity (PWV) techniques, age-adjusted reference values, and thresholds. In the Framingham Heart Study, abnormal aortic stiffness measures were present in approximately 24%–33% of community-dwelling adults with a mean age of 62 years, while the prevalence of abnormally high carotid-femoral PWV increased to 64% in men and 74% in women aged ≥70 years (2). In a community-based study from North China using age-specific baPWV thresholds, high baPWV was observed in 22.3% of men and 26.4% of women (3). More broadly, a systematic review and meta-analysis of 167 studies involving 509,743 participants from 34 countries demonstrated that both brachial-ankle and carotid-femoral PWV increase markedly with age and vary by sex, country, and geographic region, with higher age-standardized baPWV values reported in Asia than in Europe (4). These findings underscore that vascular stiffening is common in middle-aged and older populations but is strongly dependent on age, population characteristics, measurement method, and the definition used.

Obesity is another major and rapidly increasing global health burden. A pooled analysis of 3,663 population-representative studies involving 222 million participants showed that the global age-standardized prevalence of obesity increased between 1990 and 2022 from 8.8% to 18.5% in women and from 4.8% to 14.0% in men, corresponding to hundreds of millions of adults living with obesity worldwide (5). Importantly, obesity is metabolically heterogeneous, and body mass index (BMI) alone does not fully characterize cardiometabolic risk. Metabolically healthy obesity (MHO) generally refers to individuals with obesity who have relatively preserved insulin sensitivity and few or no major metabolic abnormalities, whereas metabolically obese normal weight (MONW), also termed metabolically unhealthy normal weight in some studies, describes normal-weight individuals who nevertheless exhibit insulin resistance, metabolic syndrome components, visceral or ectopic adiposity, or other adverse metabolic features (6). Although definitions vary across studies, these phenotypes have clinical relevance because apparently favorable body size or metabolic status may not imply long-term vascular health. Meta-analyses have reported that MHO is associated with a higher long-term risk of cardiovascular events compared with metabolically healthy normal-weight individuals, while metabolically abnormal normal-weight individuals also have increased risks of cardiovascular disease and mortality (7, 8). Therefore, simultaneous consideration of adiposity and metabolic health may improve identification of individuals whose vascular risk would be underestimated if BMI or conventional obesity status were considered alone.

Accumulating evidence suggests that metabolic phenotype categories are also associated with differences in vascular stiffness, although the magnitude and direction of these associations have varied according to phenotype definitions, population characteristics, and PWV methodology. In Korean adults without established cardiovascular disease, metabolically abnormal normal-weight individuals had higher baPWV than participants with MHO, suggesting that metabolic dysfunction may be particularly relevant to adverse vascular characteristics even in the absence of obesity (9). In a Chinese cohort, Yuan et al. (10) found that metabolically unhealthy non-obese and metabolically unhealthy obese phenotypes were associated with greater arterial stiffness than the metabolically healthy non-obese phenotype, with particularly notable associations in women; in the longitudinal component of that study, metabolically unhealthy phenotypes were also associated with subsequent arterial stiffening. Conversely, data from the large Kailuan cohort suggested that both BMI category and metabolic health contribute to abnormal baPWV, with MHO showing an intermediate vascular-risk pattern rather than a uniformly benign phenotype (11). More recently, a cross-sectional study of 13,435 Chinese adults reported higher baPWV in metabolically unhealthy non-obese and metabolically unhealthy obese individuals than in metabolically healthy phenotypes, whereas MHO was not independently associated with elevated baPWV after adjustment (12). Collectively, these studies support an association between metabolic phenotype and vascular stiffness but also indicate substantial heterogeneity. Moreover, age- and sex-related differences in PWV trajectories and prior evidence of sex-specific phenotype associations suggest that analyses stratified by sex and age may provide additional clinically relevant information (4, 10).

PWV is a noninvasive hemodynamic measure based on the principle that the speed of propagation of the arterial pressure wave increases as the arterial wall becomes less distensible. In general, PWV is calculated from the distance between two arterial recording sites divided by the pulse transit time between those sites. Carotid-femoral PWV (cfPWV), commonly measured using applanation tonometry or related pressure-sensor techniques at the carotid and femoral arteries, primarily reflects stiffness of the aorta and central elastic arteries and has been recommended as a reference approach for noninvasive assessment of large-artery stiffness in vascular research (13). Brachial-ankle PWV (baPWV), in contrast, is usually obtained with an automated oscillometric device using cuffs placed on both arms and ankles; it incorporates the aorta as well as peripheral muscular arterial segments and can be measured rapidly with limited operator dependence (14). Asian expert consensus recognizes baPWV as a practical measure that includes both central elastic and peripheral muscular arteries, while emphasizing the need for standardized measurement and appropriate clinical interpretation (15). PWV values are associated with cardiovascular events and mortality in observational studies, and contemporary guidelines acknowledge cfPWV and baPWV as measures that may contribute to cardiovascular risk assessment. Nevertheless, PWV—particularly baPWV—should be interpreted as a noninvasive marker of vascular stiffness and cardiovascular risk rather than as a stand-alone diagnostic standard for arterial stiffness, and threshold-based classifications may vary across devices and populations (13, 15).

Beyond its role as a research measure, PWV has potential clinical utility as an instrumental marker of vascular aging and cardiovascular risk. Contemporary European guidelines recognize arterial stiffness assessed by carotid-femoral or brachial-ankle PWV as an optional measure for identifying vascular hypertension-mediated organ damage and for refining cardiovascular risk when conventional risk assessment leaves clinical uncertainty. In Asian populations, baPWV is particularly attractive because it can be obtained rapidly and noninvasively with automated oscillometric devices and incorporates both central elastic and peripheral muscular arterial segments. Nevertheless, PWV is currently used more commonly in research and specialized clinical settings than in routine practice, and its interpretation should account for measurement technique, blood pressure, age, and population-specific thresholds.

Against this background, the present single-center cross-sectional study examined adults undergoing routine self-paid health examinations and classified participants into metabolically healthy normal weight, MHO, MONW, and metabolically unhealthy obesity phenotypes according to adiposity and metabolic status. The primary objective was to evaluate the association between metabolic phenotype categories and arterial stiffness-related vascular status, operationalized in this study using a prespecified baPWV threshold. The second objective was to determine whether these associations differed by sex, with additional age-stratified analyses, thereby assessing whether phenotype-based and sex-specific patterns may provide clinically useful information for identifying individuals who could benefit from more detailed cardiovascular risk assessment. Because of the cross-sectional design, the analyses were intended to characterize associations rather than temporal or causal relationships; similarly, elevated baPWV was treated as a vascular risk marker rather than a definitive clinical diagnosis of arterial stiffness.

## 2 Materials and methods

### 2.1 Study design and population

This cross-sectional observational study included adults aged ≥18 years who underwent routine annual self-paid health examinations at Xiamen Chang-Gung Hospital between 2014 and 2016. Following approval by the Institutional Review Board of Xiamen Chang-Gung Hospital, de-identified electronic health-examination records were retrieved for data processing and analysis. The study was conducted in accordance with the ethical principles of the Declaration of Helsinki.

A total of 19,465 individuals in the health-examination database were initially screened. Participants were required to have complete data for the variables necessary for metabolic phenotype classification, covariate adjustment, and vascular analyses. Of the initially screened individuals, 9,484 were excluded because of missing data for one or more required demographic, clinical, anthropometric, laboratory, lifestyle, medication, or vascular variables. Missing data were handled using a complete-case approach, without statistical imputation. Consequently, 9,981 participants were included in the final analysis. The participant selection process is summarized in Figure 1.

**Figure 1:**
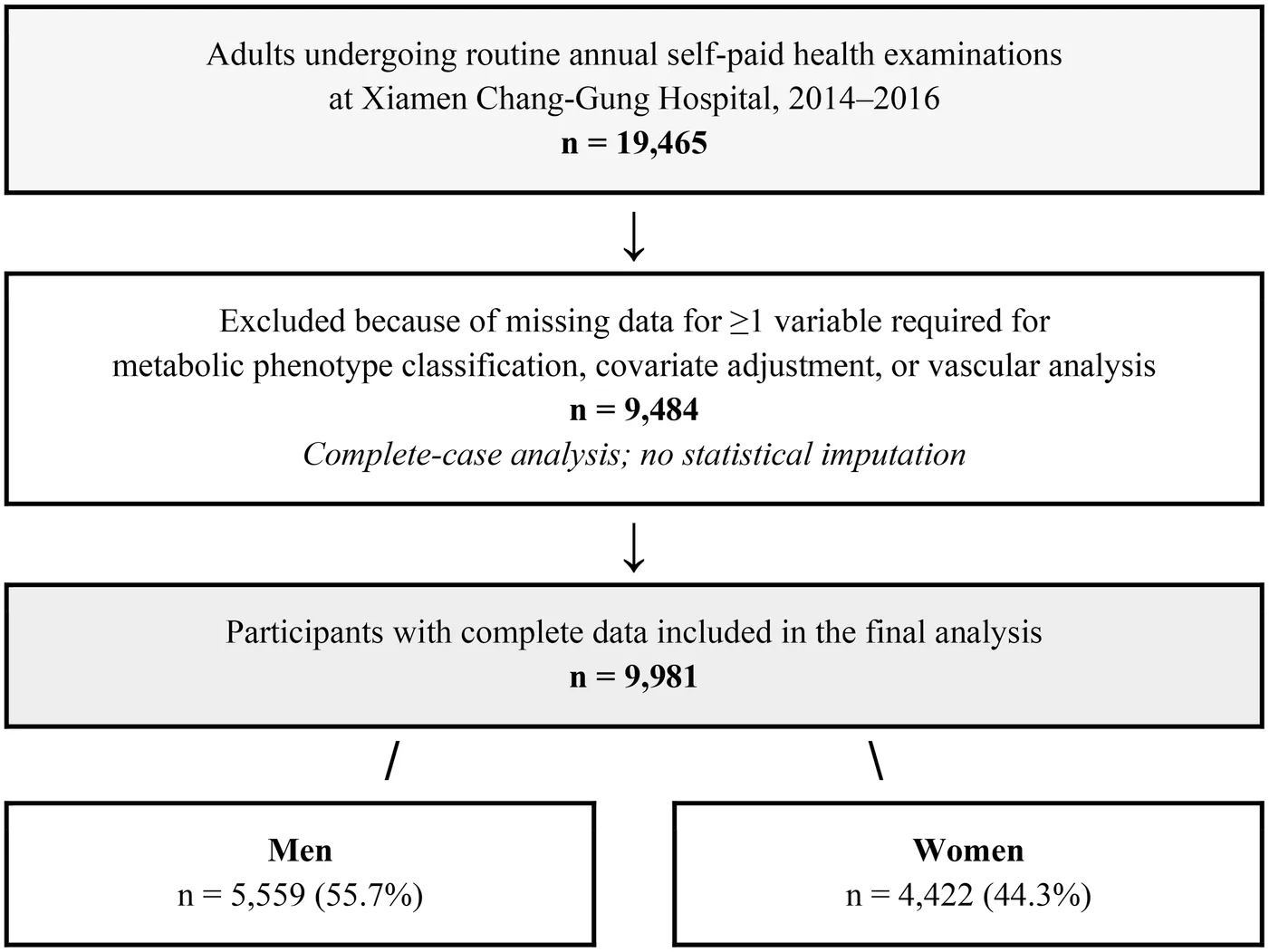
Flowchart of participant selection. A total of 19,465 individuals who underwent routine annual self-paid health examinations between 2014 and 2016 were initially screened. Of these, 9,484 were excluded because of missing data for one or more variables required for metabolic phenotype classification, covariate adjustment, or vascular analyses. The final complete-case analytic cohort consisted of 9,981 participants (5,559 men and 4,422 women). No statistical imputation of missing data was performed.

### 2.2 Eligibility criteria

Participants were eligible if they had the demographic, clinical, anthropometric, laboratory, and vascular measurements required for the present analyses, including age, sex, height, body weight, waist circumference, blood pressure, fasting plasma glucose, fasting insulin, lipid profile, smoking status, alcohol-use status, antihypertensive medication use, brachial-ankle pulse wave velocity (baPWV), and ankle-brachial index (ABI). All participants fasted for at least 12 h before blood sampling, and female participants confirmed that they were not pregnant at the time of the examination. Participants lacking information required for metabolic phenotype classification or the principal arterial stiffness analyses were excluded.

### 2.3 Data collection and laboratory measurements

Medical history and lifestyle information were obtained using a structured health examination questionnaire administered by trained personnel. Information included previous diagnoses of chronic medical conditions, current treatment status, smoking, and alcohol consumption. For the present analyses, current smoking, current alcohol use, and antihypertensive medication use were coded as binary variables. Because the data were derived from routine self-paid health examinations, detailed information on individual medication names or classes, doses, treatment duration, treatment changes, and adherence was not consistently available.

All biochemical measurements were performed in a College of American Pathologists-accredited laboratory. Fasting plasma glucose was measured using a modified hexokinase enzymatic method (Cobas Mira Chemistry System, Roche Diagnostics, USA). Total cholesterol (TC), low-density lipoprotein cholesterol (LDL-C), high-density lipoprotein cholesterol (HDL-C), and triglycerides (TG) were measured using an automated biochemical analyzer (UniCel DxC 800, Beckman Coulter, Ireland). Fasting insulin concentrations were determined by radioimmunoassay using a double-antibody method (Pharmacia Diagnostics, Sweden). Laboratory instruments were routinely calibrated in accordance with standard quality-control procedures.

### 2.4 Anthropometric and hemodynamic measurements

Height and body weight were measured to the nearest 0.1 cm and 0.1 kg, respectively. Body mass index (BMI) was calculated as body weight in kilograms divided by height in meters squared (kg/m^2^). Waist circumference was measured at the midpoint between the lower rib margin and the iliac crest. Blood pressure was measured three times after at least 15 min of seated rest using an automated sphygmomanometer, and the mean of the three measurements was used for analysis.

Mean arterial pressure (MAP) was calculated as:

MAP = (2  ×  diastolic blood pressure + systolic blood pressure)/3.

### 2.5 Definition of metabolic syndrome and insulin resistance

Metabolic syndrome (MS) was defined according to the National Cholesterol Education Program Adult Treatment Panel III criteria, with waist circumference thresholds modified for Asian populations. MS was defined by the presence of at least three of the following five components: (1) elevated blood pressure, defined as systolic blood pressure ≥130 mmHg and/or diastolic blood pressure ≥85 mmHg, current antihypertensive treatment, or a previous diagnosis of hypertension; (2) elevated TG ≥1.7 mmol/L or current treatment for hypertriglyceridemia; (3) reduced HDL-C, defined as <1.03 mmol/L in men and <1.29 mmol/L in women, or current treatment for dyslipidemia; (4) hyperglycemia, defined as fasting plasma glucose ≥5.6 mmol/L, current treatment for hyperglycemia/diabetes, or a previous diagnosis of diabetes mellitus; and (5) abdominal obesity, defined as waist circumference ≥90 cm in men and ≥80 cm in women.

Insulin resistance was estimated using the homeostasis model assessment of insulin resistance (HOMA-IR), calculated as fasting insulin (mIU/L)  ×  fasting plasma glucose (mmol/L)/22.5. A HOMA-IR value ≥2.5 was used as the prespecified operational threshold for insulin resistance. This threshold was based on previous Asian reference data showing a HOMA-IR reference interval of 0.4–2.4 in healthy adults, with values ≥2.5 considered indicative of insulin resistance. Because HOMA-IR thresholds may vary according to ethnicity and study population, this cutoff was used for phenotype classification rather than as a universal diagnostic criterion.

### 2.6 Definition of metabolic phenotypes

For the present phenotype classification, obesity was operationally defined as BMI ≥25 kg/m^2^ according to the Asia-Pacific BMI classification. This cutoff was selected for consistency with an Asian-specific adiposity framework and was used to distinguish normal-weight and obese phenotype categories. We acknowledge that country-specific thresholds differ and that Chinese national criteria commonly define obesity at a higher BMI threshold. Participants were classified into four mutually exclusive metabolic phenotypes according to BMI, HOMA-IR, and MS status: metabolically healthy normal weight (MHNW), defined as BMI <25 kg/m^2^, HOMA-IR <2.5, and absence of MS; metabolically healthy obesity (MHO), defined as BMI ≥25 kg/m^2^, HOMA-IR <2.5, and absence of MS; metabolically obese normal weight (MONW), defined as BMI <25 kg/m^2^ with HOMA-IR ≥2.5 and/or MS; and metabolically unhealthy obesity (MUO), defined as BMI ≥25 kg/m^2^ with HOMA-IR ≥2.5 and/or MS.

### 2.7 Arterial stiffness assessment

Brachial-ankle pulse wave velocity (baPWV) and ankle-brachial index (ABI) were measured using a validated oscillometric device (VP-1,000; Colin Co. Ltd., Komaki, Japan), which simultaneously recorded pulse waves and systolic blood pressure from both brachial and posterior tibial arteries. The mean of the right- and left-sided baPWV values was used for analysis. ABI was summarized as the mean of the available right- and left-sided values and was included as a descriptive vascular parameter only.

### 2.8 Definition of the primary vascular outcome

For the present analyses, elevated vascular stiffness was operationally defined as a mean bilateral baPWV >1700cm/s according to the prespecified study threshold. This binary baPWV-defined outcome was used in the logistic regression analyses. ABI was not incorporated into this outcome, and no composite baPWV/ABI definition was used.

### 2.9 Statistical analysis

The distribution of continuous variables was assessed using the Shapiro–Wilk test. Because the principal continuous variables were non-normally distributed within the relevant analytical strata, continuous variables were summarized as the median and interquartile range (IQR), whereas categorical variables were presented as numbers and percentages. For comparisons between men and women, continuous variables were analyzed using the Mann–Whitney U test and categorical variables using the chi-square test. The distributions of the four metabolic phenotypes were also compared between sexes using the chi-square test.

Participants were subsequently stratified by sex and age (<50 and ≥50 years). Within each sex- and age-specific stratum, continuous characteristics across the four metabolic phenotypes were compared using the Kruskal–Wallis test, and categorical characteristics, including elevated baPWV status, were compared using the chi-square test. When the overall Kruskal–Wallis test was statistically significant, *post hoc* pairwise comparisons were performed using the Mann–Whitney U test. Bonferroni correction was applied to the six possible pairwise comparisons among the four phenotypes, and a Bonferroni-adjusted *P* value <0.05 was considered statistically significant. Linear trends across the ordered phenotype categories (MHNW, MHO, MONW, and MUO, coded 1 through 4) were evaluated using an ordinary least-squares linear trend model with phenotype code entered as a continuous predictor. The same prespecified trend approach was applied to the binary elevated baPWV indicator.

Associations between metabolic phenotype and elevated baPWV were evaluated using binary logistic regression separately within each sex- and age-specific stratum, with MHNW as the reference category. Odds ratios (ORs) and 95% confidence intervals (CIs) were estimated using two models. Model 1 was unadjusted. Model 2 was adjusted for age as a continuous variable, current smoking, and current alcohol consumption. Continuous age was retained within the <50- and ≥50-year strata to account for residual age-related variation in baPWV. Variables used directly or indirectly to construct the metabolic phenotype, including BMI, HOMA-IR, fasting glucose, triglycerides, HDL-C, blood pressure, and antihypertensive treatment status, were not included in the primary adjusted model to avoid overadjustment.

To address multiplicity in the principal phenotype-baPWV analyses, the Benjamini-Hochberg false discovery rate (FDR) procedure was applied within each sex and each logistic regression model to the six non-reference comparisons (MHO, MONW, and MUO across the two age strata). Both raw and FDR-adjusted *P* values were calculated, with an FDR-adjusted *P* value <0.05 considered statistically significant. A prespecified phenotype-by-sex interaction analysis was additionally performed in the overall cohort. Joint interaction significance was assessed using a likelihood-ratio test comparing models with and without phenotype-by-sex interaction terms. The adjusted interaction model included age, current smoking, and current alcohol consumption. Because the interaction represented a separate prespecified hypothesis, its *P* value was evaluated independently and was not included in the FDR correction family.

Two sensitivity analyses were performed to evaluate robustness. First, because some sex- and age-specific strata contained few elevated baPWV events, particularly women aged <50 years, the adjusted phenotype analysis was repeated using Firth bias-reduced penalized logistic regression with the same Model 2 covariates (age, current smoking, and current alcohol consumption). Firth odds ratios were reported with 95% profile penalized-likelihood confidence intervals, and statistical significance was assessed using penalized likelihood-ratio tests. Second, to assess whether the findings depended on dichotomizing baPWV at 1700cm/s, mean bilateral baPWV was analyzed as a continuous outcome after natural-log transformation. Within each sex- and age-specific stratum, ordinary least-squares models adjusted for age, current smoking, and current alcohol consumption were fitted using HC3 heteroskedasticity-robust standard errors. Regression coefficients were exponentiated and expressed as adjusted percentage differences in baPWV relative to MHNW. For each sensitivity analysis, the Benjamini-Hochberg FDR procedure was applied within each sex across the six non-reference phenotype comparisons.

All statistical tests were two-sided. Except for the Bonferroni-adjusted *post hoc* comparisons and FDR-adjusted analyses described above, a *P* value <0.05 was considered statistically significant. Statistical analyses were performed using Python, primarily with the SciPy and statsmodels packages.

## 3 Results

### 3.1 Study population and sex-specific characteristics

Of the 19,465 individuals initially screened, 9,484 were excluded because of missing data for one or more variables required for the present analyses. The final complete-case analytic cohort therefore comprised 9,981 participants, including 5,559 men (55.7%) and 4,422 women (44.3%). The median age was 46.0 years [interquartile range (IQR), 40.0–54.0] in men and 47.0 years (IQR, 40.0–55.0) in women (*P* = 0.004). Compared with women, men had significantly higher body mass index (BMI), waist circumference, mean arterial pressure (MAP), fasting glucose, total cholesterol, triglycerides, low-density lipoprotein cholesterol, brachial-ankle pulse wave velocity (baPWV), ankle-brachial index (ABI), insulin, and homeostasis model assessment of insulin resistance values, whereas women had higher high-density lipoprotein cholesterol levels (all *P* < 0.01). Elevated baPWV was present in 748 participants (7.5%), including 396 men (7.1%) and 352 women (8.0%), with no significant difference between sexes (*P* = 0.115). The distribution of metabolic phenotypes differed significantly between men and women (*P* < 0.001). MHNW was more frequent in women than in men (67.1% vs. 50.4%), whereas MHO and MUO were more frequent in men (18.8% vs. 12.5% and 23.4% vs. 12.3%, respectively). MONW accounted for 7.3% of men and 8.0% of women (Table 1).

**Table 1:** Main characteristics of the study subjects by gender.

Characteristics	Total	Men	Women	*P* value
Number of subjects	9,981	5,559	4,422	
Age, years	46.0 (40.0–54.0)	46.0 (40.0–54.0)	47.0 (40.0–55.0)	0.004
BMI (kg/m^2^)	23.69 (21.53–25.95)	24.41 (22.36–26.47)	22.71 (20.75–24.97)	<0.001
Waist circumference (cm)	83.0 (76.0–90.0)	87.0 (81.0–92.0)	78.0 (72.0–84.0)	<0.001
Mean arterial pressure (mmHg)	86.0 (77.7–95.3)	88.7 (81.0–97.7)	82.0 (74.0–91.7)	<0.001
Fasting glucose (mmol/L)	5.05 (4.75–5.43)	5.10 (4.79–5.50)	4.99 (4.71–5.34)	<0.001
Total cholesterol (mmol/L)	5.13 (4.53–5.80)	5.21 (4.60–5.85)	5.05 (4.45–5.73)	<0.001
Triglycerides (mmol/L)	1.20 (0.84–1.81)	1.42 (1.00–2.11)	0.98 (0.72–1.40)	<0.001
LDL cholesterol (mmol/L)	3.26 (2.73–3.84)	3.37 (2.83–3.95)	3.11 (2.60–3.69)	<0.001
HDL cholesterol (mmol/L)	1.27 (1.09–1.50)	1.17 (1.02–1.36)	1.42 (1.23–1.63)	<0.001
baPWV (cm/s)	1,292.0 (1,178.5–1,442.0)	1,317.5 (1,215.0–1,452.5)	1,251.5 (1,131.1–1,422.9)	<0.001
ABI	1.125 (1.070–1.180)	1.140 (1.085–1.195)	1.110 (1.060–1.160)	<0.001
Insulin (mIU/L)	6.00 (4.40–8.40)	6.10 (4.30–8.60)	5.90 (4.40–8.10)	0.003
HOMA-IR	1.37 (0.96–2.01)	1.44 (0.98–2.11)	1.32 (0.95–1.90)	<0.001
Arterial stiffness, *n* (%)	748 (7.5%)	396 (7.1%)	352 (8.0%)	0.115
MHNW, *n* (%)	5,771 (57.8%)	2,803 (50.4%)	2,968 (67.1%)	<0.001
MHO, *n* (%)	1,599 (16.0%)	1,046 (18.8%)	553 (12.5%)	
MONW, *n* (%)	762 (7.6%)	407 (7.3%)	355 (8.0%)	
MUO, *n* (%)	1,849 (18.5%)	1,303 (23.4%)	546 (12.3%)	

Values ​​are median(IQR) unless otherwise indicated. Continuous variables were compared using the Mann–Whitney U test; categorical variables using the chi-square test. The phenotype P value is the omnibus sex-by-phenotype chi-square test and is displayed on the MHNW row.

### 3.2 Metabolic phenotype characteristics in men

Among men, progressively less favorable metabolic phenotypes were generally characterized by higher adiposity, blood pressure, fasting glucose, triglycerides, and baPWV, together with lower HDL-C (Table 2). In men aged <50 years, elevated baPWV prevalence was 0.8% in MHNW, 1.1% in MHO, 6.3% in MONW, and 3.8% in MUO (overall *P* < 0.001). Among men aged ≥50 years, the corresponding prevalences were 12.3%, 9.9%, 27.2%, and 22.3% (overall *P* < 0.001). Significant trends were observed for most cardiometabolic variables and baPWV; notable exceptions included the absence of a significant ABI trend in men aged <50 years and no significant between-phenotype difference in total cholesterol among men aged ≥50 years. Detailed numerical values and *post hoc* comparisons are presented in Table 2.

**Table 2:** Baseline characteristics of men according to metabolic phenotypes stratified by age.

Characteristic	MHNW	MHO	MONW	MUO	*P* value	*P* for trend
Men <50 years (*n* = 3,459)						
Number	1,716	724	205	814		
Age, years	41.0 (37.0–45.0)	42.0 (38.0–45.0)	42.0 (39.0–46.0)	42.0 (38.0–46.0)	0.008	0.004
BMI (kg/m^2^)	22.56 (20.94–23.76)	26.38 (25.62–27.49)	23.93 (23.08–24.55)	27.95 (26.47–29.50)	<0.001	<0.001
Waist circumference (cm)	81.0 (77.0–85.0)	90.0 (87.0–94.0)	86.0 (83.0–90.0)	95.0 (91.0–99.0)	<0.001	<0.001
Mean arterial pressure (mmHg)	83.7 (77.3–91.0)	87.5 (81.9–94.3)	93.7 (86.3–102.7)	97.7 (87.3–105.0)	<0.001	<0.001
Fasting glucose (mmol/L)	4.91 (4.64–5.18)	5.00 (4.75–5.26)	5.53 (5.10–6.36)	5.38 (5.00–5.93)	<0.001	<0.001
Total cholesterol (mmol/L)	5.07 (4.55–5.70)	5.25 (4.67–5.87)	5.30 (4.71–5.97)	5.44 (4.79–6.13)	<0.001	<0.001
Triglycerides (mmol/L)	1.18 (0.87–1.70)	1.45 (1.10–1.88)	2.23 (1.55–3.37)	2.25 (1.71–3.21)	<0.001	<0.001
LDL cholesterol (mmol/L)	3.27 (2.78–3.80)	3.50 (2.96–4.07)	3.36 (2.83–3.92)	3.46 (2.90–4.15)	<0.001	<0.001
HDL cholesterol (mmol/L)	1.26 (1.10–1.47)	1.16 (1.06–1.30)	1.01 (0.91–1.19)	1.04 (0.94–1.19)	<0.001	<0.001
baPWV (cm/s)	1,264.8 (1,178.0–1,361.6)	1,270.8 (1,183.9–1,353.6)	1,339.0 (1,245.5–1,469.0)	1,320.8 (1,216.5–1,443.1)	<0.001	<0.001
ABI	1.125 (1.070–1.170)	1.140 (1.085–1.200)	1.125 (1.065–1.175)	1.140 (1.080–1.195)	<0.001	0.418
Arterial stiffness, *n* (%)	14 (0.8%)	8 (1.1%)	13 (6.3%)	31 (3.8%)	<0.001	<0.001
Men ≥50 years (*n* = 2,100)						
Number	1,087	322	202	489		
Age, years	58.0 (53.0–62.0)	57.0 (52.0–61.0)	56.0 (52.0–62.0)	56.0 (52.0–62.0)	0.032	0.068
BMI (kg/m^2^)	22.46 (20.77–23.64)	26.23 (25.59–27.24)	23.84 (22.89–24.50)	27.00 (26.04–28.38)	<0.001	<0.001
Waist circumference (cm)	82.0 (77.0–86.0)	91.0 (88.0–95.0)	88.0 (84.0–91.0)	94.0 (91.0–99.0)	<0.001	<0.001
Mean arterial pressure (mmHg)	86.7 (79.7–94.7)	90.3 (83.3–97.7)	95.3 (86.7–103.6)	98.0 (90.3–106.3)	<0.001	<0.001
Fasting glucose (mmol/L)	5.06 (4.77–5.38)	5.11 (4.87–5.39)	5.87 (5.25–6.77)	5.78 (5.19–6.78)	<0.001	<0.001
Total cholesterol (mmol/L)	5.17 (4.55–5.84)	5.26 (4.68–5.73)	5.33 (4.73–5.94)	5.17 (4.51–5.95)	0.271	0.262
Triglycerides (mmol/L)	1.10 (0.81–1.46)	1.25 (0.94–1.56)	2.03 (1.47–2.75)	2.00 (1.43–2.74)	<0.001	<0.001
LDL cholesterol (mmol/L)	3.33 (2.81–3.94)	3.54 (3.01–4.02)	3.35 (2.78–3.98)	3.40 (2.75–4.00)	0.006	0.875
HDL cholesterol (mmol/L)	1.26 (1.10–1.47)	1.19 (1.06–1.32)	1.01 (0.89–1.19)	1.01 (0.92–1.18)	<0.001	<0.001
baPWV (cm/s)	1,377.5 (1,261.2–1,537.0)	1,370.0 (1,251.0–1,524.4)	1,483.8 (1,355.1–1,725.8)	1,487.0 (1,325.0–1,685.5)	<0.001	<0.001
ABI	1.155 (1.100–1.208)	1.165 (1.110–1.220)	1.155 (1.096–1.214)	1.170 (1.115–1.215)	0.010	0.018
Arterial stiffness, *n* (%)	134 (12.3%)	32 (9.9%)	55 (27.2%)	109 (22.3%)	<0.001	<0.001

*P* values for continuous variables were obtained using the Kruskal–Wallis test; arterial stiffness was compared using the chi-square test. *P* for trend was obtained from an ordinary least-squares trend model with phenotype coded 1–4. When the omnibus Kruskal–Wallis test was significant, six pairwise Mann–Whitney U tests were performed with Bonferroni adjustment.

### 3.3 Metabolic phenotype characteristics in women

A similar overall pattern was observed among women, with metabolically unhealthy phenotypes generally showing less favorable cardiometabolic profiles and higher baPWV (Table 3). In women aged <50 years, elevated baPWV prevalence was 0.2% in MHNW, 1.2% in MHO, 2.3% in MONW, and 2.4% in MUO (overall *P* < 0.001). Among women aged ≥50 years, the corresponding prevalences were 12.8%, 12.5%, 30.4%, and 28.3% (overall *P* < 0.001). Most cardiometabolic variables differed significantly across phenotypes; however, total cholesterol did not differ significantly among women aged ≥50 years, and the linear trends for BMI and LDL-C were not significant in this stratum. Detailed numerical values and *post hoc* comparisons are presented in Table 3.

**Table 3:** Baseline characteristics of women according to metabolic phenotypes stratified by age.

Characteristic	MHNW	MHO	MONW	MUO	*P* value	*P* for trend
Women <50 years (*n* = 2,550)						
Number	2,013	241	131	165		
Age, years	41.0 (36.0–45.0)	45.0 (40.0–47.0)	44.0 (40.0–47.0)	44.0 (40.0–46.0)	<0.001	<0.001
BMI (kg/m^2^)	21.18 (19.73–22.68)	26.27 (25.53–27.53)	23.14 (22.09–24.06)	27.32 (26.05–29.22)	<0.001	<0.001
Waist circumference (cm)	73.0 (69.0–78.0)	85.0 (81.0–89.0)	79.0 (74.5–84.0)	88.0 (85.0–92.0)	<0.001	<0.001
Mean arterial pressure (mmHg)	76.3 (70.7–83.0)	82.7 (74.0–91.3)	85.7 (77.3–95.8)	87.7 (81.0–98.0)	<0.001	<0.001
Fasting glucose (mmol/L)	4.84 (4.61–5.09)	4.94 (4.71–5.21)	5.50 (5.08–5.76)	5.37 (5.05–5.92)	<0.001	<0.001
Total cholesterol (mmol/L)	4.76 (4.25–5.32)	5.00 (4.41–5.66)	5.05 (4.49–5.62)	4.99 (4.37–5.65)	<0.001	<0.001
Triglycerides (mmol/L)	0.80 (0.62–1.06)	1.06 (0.83–1.31)	1.52 (0.98–2.12)	1.69 (1.14–2.25)	<0.001	<0.001
LDL cholesterol (mmol/L)	2.83 (2.42–3.35)	3.16 (2.66–3.75)	3.18 (2.73–3.62)	3.22 (2.74–3.67)	<0.001	<0.001
HDL cholesterol (mmol/L)	1.48 (1.31–1.68)	1.38 (1.24–1.55)	1.25 (1.08–1.43)	1.15 (1.03–1.28)	<0.001	<0.001
baPWV (cm/s)	1,153.5 (1,072.5–1,245.0)	1,189.0 (1,097.5–1,300.0)	1,262.0 (1,143.8–1,385.2)	1,283.0 (1,176.0–1,401.0)	<0.001	<0.001
ABI	1.085 (1.040–1.135)	1.120 (1.065–1.170)	1.095 (1.042–1.132)	1.110 (1.060–1.160)	<0.001	<0.001
Arterial stiffness, *n* (%)	5 (0.2%)	3 (1.2%)	3 (2.3%)	4 (2.4%)	<0.001	<0.001
Women ≥50 years (*n* = 1,872)						
Number	955	312	224	381		
Age, years	56.0 (52.0–60.0)	56.0 (52.8–61.0)	58.0 (54.0–62.0)	58.0 (54.0–63.0)	<0.001	<0.001
BMI (kg/m^2^)	22.16 (20.73–23.48)	26.41 (25.61–27.46)	23.64 (22.48–24.31)	27.23 (26.03–29.17)	<0.001	0.109
Waist circumference (cm)	77.0 (73.0–81.5)	86.0 (83.0–90.0)	83.0 (78.0–86.0)	90.0 (87.0–96.0)	<0.001	<0.001
Mean arterial pressure (mmHg)	85.3 (77.2–93.0)	89.5 (81.2–98.3)	94.5 (86.3–102.4)	96.0 (88.7–103.3)	<0.001	<0.001
Fasting glucose (mmol/L)	5.02 (4.75–5.30)	5.04 (4.79–5.32)	5.79 (5.30–6.37)	5.61 (5.16–6.33)	<0.001	<0.001
Total cholesterol (mmol/L)	5.39 (4.79–6.10)	5.47 (4.85–6.15)	5.47 (4.79–6.12)	5.44 (4.81–6.10)	0.764	0.742
Triglycerides (mmol/L)	0.98 (0.75–1.31)	1.15 (0.88–1.42)	1.77 (1.25–2.32)	1.77 (1.30–2.29)	<0.001	<0.001
LDL cholesterol (mmol/L)	3.37 (2.83–3.90)	3.51 (2.98–4.05)	3.38 (2.91–4.06)	3.49 (2.96–4.09)	0.032	0.124
HDL cholesterol (mmol/L)	1.47 (1.30–1.72)	1.45 (1.32–1.62)	1.23 (1.11–1.39)	1.18 (1.07–1.33)	<0.001	<0.001
baPWV (cm/s)	1,354.5 (1,242.2–1,522.0)	1,404.5 (1,257.0–1,550.0)	1,558.8 (1,398.8–1,736.4)	1,521.5 (1,401.5–1,730.5)	<0.001	<0.001
ABI	1.130 (1.085–1.175)	1.140 (1.094–1.185)	1.143 (1.105–1.190)	1.140 (1.095–1.190)	0.004	<0.001
Arterial stiffness, *n* (%)	122 (12.8%)	39 (12.5%)	68 (30.4%)	108 (28.3%)	<0.001	<0.001

*P* values for continuous variables were obtained using the Kruskal–Wallis test; arterial stiffness was compared using the chi-square test. *P* for trend was obtained from an ordinary least-squares trend model with phenotype coded 1–4. When the omnibus Kruskal–Wallis test was significant, six pairwise Mann–Whitney U tests were performed with Bonferroni adjustment.

### 3.4 Associations between metabolic phenotypes and elevated baPWV

In logistic regression analyses using MHNW as the reference category, MONW and MUO were associated with elevated baPWV across both age strata in men (Table 4). Among men aged <50 years, MONW was strongly associated with elevated baPWV in the unadjusted model (OR, 8.23; 95% CI, 3.81–17.77; *P* < 0.001) and remained significant after adjustment for age, smoking, and alcohol consumption (Model 2: OR, 7.68; 95% CI, 3.55–16.62; *P* < 0.001). MUO also remained associated with elevated baPWV in Model 2 (OR, 4.61; 95% CI, 2.44–8.74; *P* < 0.001), whereas MHO was not (OR, 1.32; 95% CI, 0.55–3.17; *P* = 0.531). Among men aged ≥50 years, both MONW and MUO remained associated with elevated baPWV in Model 2 (MONW: OR, 3.37; 95% CI, 2.29–4.96; *P* < 0.001; MUO: OR, 2.34; 95% CI, 1.73–3.17; *P* < 0.001), whereas MHO was not significantly associated with the outcome (OR, 0.85; 95% CI, 0.55–1.30; *P* = 0.448).

**Table 4:** Unadjusted and adjusted odds ratios for elevated baPWV by metabolic phenotype.

Group/phenotype	Elevated baPWV, *n* (%)	Model 1 OR (95% CI)	*P* value	Model 2 OR (95% CI)	*P* value	Model 2 FDR P
Men <50 years						
MHNW	14 (0.8%)	1.00 (reference)	—	1.00 (reference)	—	—
MHO	8 (1.1%)	1.36 (0.57–3.25)	0.492	1.32 (0.55–3.17)	0.531	0.531
MONW	13 (6.3%)	8.23 (3.81–17.77)	<0.001	7.68 (3.55–16.62)	<0.001	<0.001
MUO	31 (3.8%)	4.81 (2.55–9.10)	<0.001	4.61 (2.44–8.74)	<0.001	<0.001
Men ≥50 years						
MHNW	134 (12.3%)	1.00 (reference)	—	1.00 (reference)	—	—
MHO	32 (9.9%)	0.78 (0.52–1.18)	0.244	0.85 (0.55–1.30)	0.448	0.531
MONW	55 (27.2%)	2.66 (1.86–3.81)	<0.001	3.37 (2.29–4.96)	<0.001	<0.001
MUO	109 (22.3%)	2.04 (1.54–2.70)	<0.001	2.34 (1.73–3.17)	<0.001	<0.001
Women <50 years^†^						
MHNW	5 (0.2%)	1.00 (reference)	—	1.00 (reference)	—	—
MHO	3 (1.2%)	5.06 (1.20–21.32)	0.027	2.94 (0.69–12.61)	0.146	0.175
MONW	3 (2.3%)	9.41 (2.22–39.83)	0.002	6.12 (1.42–26.42)	0.015	0.023
MUO	4 (2.4%)	9.98 (2.65–37.52)	<0.001	6.60 (1.72–25.32)	0.006	0.012
Women ≥50 years						
MHNW	122 (12.8%)	1.00 (reference)	—	1.00 (reference)	—	—
MHO	39 (12.5%)	0.98 (0.66–1.43)	0.899	0.92 (0.61–1.39)	0.688	0.688
MONW	68 (30.4%)	2.98 (2.11–4.19)	<0.001	2.58 (1.77–3.77)	<0.001	<0.001
MUO	108 (28.3%)	2.70 (2.02–3.62)	<0.001	2.36 (1.71–3.25)	<0.001	<0.001

Reference category: MHNW. Model 1: unadjusted. Model 2: adjusted for age (continuous), current smoking, and current alcohol consumption. FDR: Benjamini-Hochberg correction within each sex across six non-reference comparisons (MHO, MONW, and MUO across the two age strata).

† The conventional maximum-likelihood Model 2 in women aged <50 years generated a convergence warning; Firth penalized logistic regression is reported in Supplementary Table S1. Elevated baPWV was operationally defined as mean bilateral baPWV >1,700cm/s.

Among women aged <50 years, MHO, MONW, and MUO were all significantly associated with elevated baPWV in the unadjusted model; after adjustment for age, smoking, and alcohol consumption, MONW (OR, 6.12; 95% CI, 1.42–26.42; *P* = 0.015) and MUO (OR, 6.60; 95% CI, 1.72–25.32; *P* = 0.006) remained significant, whereas MHO did not (OR, 2.94; 95% CI, 0.69–12.61; *P* = 0.146). Among women aged ≥50 years, both MONW and MUO remained associated with elevated baPWV in Model 2 (MONW: OR, 2.58; 95% CI, 1.77–3.77; *P* < 0.001; MUO: OR, 2.36; 95% CI, 1.71–3.25; *P* < 0.001), whereas MHO was not significantly associated with the outcome (OR, 0.92; 95% CI, 0.61–1.39; *P* = 0.688).

### 3.5 Multiplicity-adjusted and interaction analyses

After Benjamini-Hochberg FDR correction, the Model 2 associations of MONW and MUO with elevated baPWV remained significant in all four sex- and age-specific strata. For MONW, the FDR-adjusted *P* values were <0.001 in men aged <50 years, <0.001 in men aged ≥50 years, 0.023 in women aged <50 years, and <0.001 in women aged ≥50 years. For MUO, the corresponding FDR-adjusted *P* values were <0.001, <0.001, 0.012, and <0.001, respectively. MHO was not significantly associated with elevated baPWV after FDR correction in any stratum (FDR-adjusted *P* values: 0.531 in men aged <50 years, 0.531 in men aged ≥50 years, 0.175 in women aged <50 years, and 0.688 in women aged ≥50 years).

The unadjusted phenotype-by-sex interaction was statistically significant (likelihood-ratio *χ*^2^ = 32.20, 3 df; *P* < 0.001). However, after adjustment for age, smoking, and alcohol consumption, the joint phenotype-by-sex interaction was no longer significant (likelihood-ratio *χ*^2^ = 2.39, 3 df; *P* = 0.495). The Model 2 term-specific interaction *P* values were 0.639 for MHO, 0.198 for MONW, and 0.934 for MUO. Thus, although the magnitudes of the phenotype associations differed numerically across sex- and age-specific strata, the adjusted interaction analysis did not provide evidence of independent effect modification by sex.

### 3.6 Sensitivity analyses

Firth penalized logistic regression yielded estimates that were closely aligned with the primary adjusted logistic model (Supplementary Table S1). MONW and MUO remained associated with higher odds of elevated baPWV in all four sex- and age-specific strata, whereas MHO remained non-significant. In women aged <50 years, the Firth estimates were OR 6.45 (95% profile CI, 1.46–24.93; *P* = 0.017; FDR-adjusted *P* = 0.025) for MONW and OR 6.59 (95% profile CI, 1.72–23.91; *P* = 0.008; FDR-adjusted *P* = 0.015) for MUO; MHO remained non-significant (OR, 3.12; 95% profile CI, 0.71–11.98; *P* = 0.123; FDR-adjusted *P* = 0.148). The conventional maximum-likelihood Model 2 in this subgroup generated a convergence warning, whereas the Firth model converged. Nevertheless, the confidence intervals remained wide, indicating substantial imprecision in the magnitude of these estimates.

The continuous-outcome sensitivity analysis also supported the overall pattern without relying on the >1700cm/s threshold (Supplementary Table S2). Relative to MHNW, adjusted baPWV was higher for MONW and MUO, respectively, by 6.64% and 4.39% in men aged <50 years, 9.98% and 7.38% in men aged ≥50 years, 7.82% and 8.38% in women aged <50 years, and 9.85% and 9.08% in women aged ≥50 years; all corresponding FDR-adjusted *P* values were <0.001. MHO was not significantly associated with continuous baPWV in any stratum. Thus, the principal pattern was not dependent solely on the prespecified dichotomous baPWV threshold.

## 4 Discussion

A distinctive feature of this study is the simultaneous evaluation of four metabolic phenotype categories in relation to baPWV-defined vascular stiffness status across sex- and age-specific strata within a relatively large health-examination cohort. After adjustment for age, smoking, and alcohol consumption, MONW and MUO were associated with higher odds of elevated baPWV in men and women in both age strata, whereas MHO was not significantly associated with the outcome. These associations for MONW and MUO remained significant after FDR correction. The adjusted phenotype-by-sex interaction was not significant (*P* = 0.495), indicating that numerical differences between sex-stratified estimates should not be interpreted as evidence of independent effect modification by sex. Two complementary sensitivity analyses supported the overall phenotype pattern: Firth penalized logistic regression yielded directionally concordant estimates despite sparse events, and analysis of continuous baPWV showed higher adjusted baPWV for MONW and MUO but not MHO across all four strata. These findings suggest that metabolic abnormalities may provide clinically relevant information when considered together with BMI, but they do not establish that metabolic status has a stronger effect than BMI itself. Because of the cross-sectional design, the observed associations do not establish temporality or causality, and baPWV should be interpreted as a noninvasive marker of vascular stiffness rather than a stand-alone diagnostic test for arterial stiffness (13, 15).

From a clinical perspective, PWV may also have value as a repeatable instrumental biomarker of the mechanical vascular profile. Beyond its established association with cardiovascular outcomes and its potential contribution to risk refinement, serial PWV assessment may provide information regarding changes in vascular function accompanying modification of cardiovascular risk factors. Supporting this concept, Bosco et al. recently evaluated 301 genetically confirmed patients with familial hypercholesterolemia receiving PCSK9 inhibitor therapy in addition to high-intensity statins and ezetimibe. After six months, reductions in LDL-C and lipoprotein(a) were accompanied by a 22.7% decrease in PWV, and participants with the greatest combined reductions in LDL-C and lipoprotein(a) showed the strongest association with PWV improvement (16). These real-world observations illustrate the potential responsiveness of PWV to intensive risk-factor modification and support its role as a mechanical vascular biomarker that may complement conventional biochemical risk assessment. However, that study assessed carotid-femoral PWV rather than baPWV and was observational; therefore, its findings should not be interpreted as evidence that PWV improvement represents regression of atherosclerotic plaque or directly translates into improved cardiovascular outcomes. Similarly, the present cross-sectional findings support the potential utility of baPWV for vascular phenotyping but do not establish its value for monitoring treatment response.

Our findings are broadly consistent with previous longitudinal studies indicating that metabolic unhealthiness is associated with adverse arterial stiffness irrespective of obesity status. In a prospective Chinese cohort, Yuan et al. reported that metabolically unhealthy non-obese and metabolically unhealthy obese phenotypes were associated with subsequent arterial stiffening, whereas MHO was not significantly associated with progression of arterial stiffness (10). This pattern is similar to our observation that MONW and MUO, but not MHO, were associated with elevated baPWV. However, longitudinal evidence is not uniform. In the Kailuan cohort, both MHO and metabolically unhealthy obesity were associated with incident arterial stiffness during follow-up, although the association was stronger for the metabolically unhealthy obese phenotype (11). Thus, our null association for MHO should not be interpreted as evidence that MHO is uniformly benign. Differences among studies may reflect heterogeneity in metabolic-health definitions, BMI thresholds, population characteristics, baseline vascular status, follow-up duration, and covariate selection. Our phenotype definition additionally incorporated HOMA-IR and metabolic syndrome status, which may identify groups that are not directly comparable with phenotypes defined using other criteria. Importantly, unlike these longitudinal cohorts, the present study is cross-sectional and therefore cannot determine whether MONW or MUO precedes vascular stiffening; the consistency with some prospective observations provides epidemiological context rather than evidence of causality.

Cross-sectional evidence also supports the relevance of metabolic health to vascular stiffness. Yoo et al. reported less favorable vascular characteristics among metabolically abnormal normal-weight adults than among individuals with MHO, whereas Guo et al. found higher baPWV among metabolically unhealthy non-obese and metabolically unhealthy obese Chinese adults but no independent association between MHO and elevated baPWV (9, 12). Data from the UK Biobank likewise showed that both excess adiposity and metabolic abnormalities were associated with higher arterial stiffness index values, underscoring that vascular phenotype may reflect contributions from both body size and metabolic status (17). These observations are compatible with our finding that metabolic abnormalities can coexist with normal BMI and adverse vascular stiffness. Potential pathways involving visceral or ectopic adiposity, insulin resistance, endothelial dysfunction, oxidative stress, inflammation, and vascular remodeling have been proposed (1, 6, 18–20), but these mechanisms were not directly measured in the present study and are therefore presented only as biologically plausible explanations rather than demonstrated causal pathways.

Recent evidence from individuals at particularly high cardiovascular risk further supports the potential relevance of metabolic profiling to vascular risk characterization. Bosco et al. evaluated 211 genetically confirmed patients with familial hypercholesterolemia receiving lipid-lowering therapy and found that those with imaging-defined subclinical atherosclerosis had a less favorable metabolic profile than those without subclinical atherosclerosis, including higher BMI, TG/HDL ratio, and TyG index. The subclinical atherosclerosis group also had a greater cumulative LDL-C burden and a more adverse innate immune profile (16). Although their vascular outcome, defined by coronary artery calcium and/or carotid or femoral plaques, differs from the baPWV-based vascular stiffness outcome evaluated in our study, their findings provide complementary evidence that metabolic abnormalities may convey clinically relevant vascular information beyond conventional assessment of adiposity or LDL-C alone. This concept is compatible with our observation that MONW was associated with elevated baPWV despite a normal BMI, whereas MHO was not independently associated with the outcome. However, because both studies are cross-sectional, these observations should be regarded as associations and do not establish that an adverse metabolic profile prospectively predicts cardiovascular events or causes subsequent vascular injury.

The sex- and age-stratified analyses provide additional context for these associations. In Model 2, MONW was associated with higher odds of elevated baPWV in men aged <50 years (OR, 7.68; 95% CI, 3.55–16.62), men aged ≥50 years (OR, 3.37; 95% CI, 2.29–4.96), women aged <50 years (OR, 6.12; 95% CI, 1.42–26.42), and women aged ≥50 years (OR, 2.58; 95% CI, 1.77–3.77). MUO was also associated with higher odds of elevated baPWV in all four strata, and all MONW and MUO associations remained significant after FDR correction. However, these subgroup estimates should not be compared solely according to their *P* values or point estimates. The formal adjusted phenotype-by-sex interaction was not significant, and no formal evidence of sex effect modification was demonstrated. In addition, the women aged <50 years subgroup contained only 15 elevated baPWV events. The conventional maximum-likelihood model generated a convergence warning in this subgroup, whereas the Firth sensitivity model converged and yielded similar MONW and MUO estimates. Although these penalized estimates remained statistically significant, their confidence intervals were wide; therefore, the apparent magnitude of the associations in younger women should be regarded as imprecise and exploratory. The continuous-baPWV sensitivity analysis produced a concordant pattern across strata, reducing concern that the overall findings were driven solely by the >1700cm/s dichotomization. Previous studies have reported sex-related differences in PWV and metabolic vascular risk; however, our adjusted interaction analysis did not demonstrate independent sex-specific effect modification (4, 10, 21–24). Because menopausal status, hormone therapy, and sex hormone levels were unavailable, the age cutoff of 50 years was used solely for age stratification and not as a surrogate for menopausal status. Therefore, estrogen- or menopause-related mechanisms remain hypotheses rather than explanations established by our data.

This study has several strengths. The relatively large sample of 9,981 adults allowed simultaneous evaluation of four metabolic phenotypes and prespecified sex- and age-stratified analyses. Participants underwent standardized health examinations, laboratory testing, anthropometric assessment, and baPWV/ABI measurement, and phenotype classification incorporated BMI, HOMA-IR, and metabolic syndrome rather than BMI alone. The primary multivariable model adjusted for age, smoking, and alcohol consumption while deliberately excluding variables used directly or indirectly to define the metabolic phenotypes, thereby reducing the risk of overadjustment. The analyses also incorporated Bonferroni-adjusted *post hoc* testing, FDR correction, a formal phenotype-by-sex interaction test, Firth penalized logistic regression to assess sparse-event bias, and a continuous-baPWV sensitivity analysis to evaluate dependence on the prespecified binary threshold. Several limitations should also be acknowledged. First, the cross-sectional design precludes determination of temporality or causality. Second, this was a single-center study of Chinese adults undergoing self-paid health examinations, which may limit generalizability and introduce selection and socioeconomic biases. Third, smoking and alcohol consumption were recorded primarily as binary variables, while detailed dietary intake, physical activity, cardiorespiratory fitness, and other lifestyle measures were unavailable; residual lifestyle-related confounding therefore remains possible. Fourth, detailed medication classes, doses, treatment duration, treatment changes, and adherence were not consistently available. Fifth, sex hormone concentrations and menopausal status were unavailable. Sixth, blood pressure was obtained during a single examination visit, albeit from repeated measurements at that visit. Seventh, baPWV reflects both central and peripheral arterial segments, is influenced by contemporaneous blood pressure and vascular tone, and is not a direct measure of atherosclerotic plaque or luminal stenosis; carotid-femoral PWV, central hemodynamic assessment, and vascular imaging were unavailable for corroboration (13–15). Although the continuous-baPWV sensitivity analysis reduced dependence on the >1700cm/s cutoff, the primary binary outcome remains an operational rather than universal diagnostic threshold. Eighth, no universally accepted definition of MHO or MONW exists, and the selected BMI, HOMA-IR, and metabolic syndrome criteria may influence phenotype classification. Finally, sparse outcome events remained an important limitation in younger women. The conventional maximum-likelihood Model 2 generated a convergence warning in women aged <50 years; Firth penalization produced convergent and directionally consistent estimates, but the wide confidence intervals demonstrate that the subgroup effect sizes remain imprecise and require confirmation in larger cohorts.

In conclusion, in this single-center cross-sectional health-examination cohort, MONW and MUO were associated with higher odds of elevated baPWV across all four sex- and age-stratified analyses after adjustment for age, smoking, and alcohol consumption, whereas MHO was not significantly associated with the outcome. Firth penalized logistic regression and continuous-baPWV sensitivity analyses yielded a concordant overall pattern, although estimates in younger women remained imprecise because of sparse events. The adjusted phenotype-by-sex interaction was not significant, and the present data therefore do not support independent effect modification by sex. These findings suggest that considering metabolic health together with BMI may provide additional information when characterizing vascular risk, but they do not establish that metabolic status has a stronger effect than BMI itself. The results are cross-sectional associations only and should not be interpreted as establishing causality or as validating baPWV >1700cm/s as a stand-alone diagnostic criterion for arterial stiffness. Multicenter longitudinal studies with larger numbers of events, repeated PWV measurements, complementary vascular assessments, and more detailed lifestyle and medication data are needed to confirm the temporal and clinical significance of these observations.

## Data Availability

The datasets presented in this article are not readily available because the data comes from the physical examination database of Xiamen Chang Gung Hospital. Because it involves basic personal information, the database cannot provide a public link to access the data. Access to the data required for the research, after removing personal data, requires approval from the IRB. Requests to access the datasets should be directed to Wen-Cheng Li, wcli3135@gmail.com.

## References

[1] Lacolley P Regnault V Laurent S. Mechanisms of arterial stiffening: from mechanotransduction to epigenetics. Arterioscler Thromb Vasc Biol. (2020) 40:1055–62. 10.1161/ATVBAHA.119.313129 32075419

[2] Mitchell GF Guo C-Y Benjamin EJ Larson MG Keyes MJ Vita JA. Cross-sectional correlates of increased aortic stiffness in the community: the framingham heart study. Circulation. (2007) 115:2628–36. 10.1161/CIRCULATIONAHA.106.667733 17485578

[3] Wang JW Zhou ZQ Hu DY. Prevalence of arterial stiffness in north China, and associations with risk factors of cardiovascular disease: a community-based study. BMC Cardiovasc Disord. (2012) 12:119. 10.1186/1471-2261-12-119 23217203 PMC3523088

[4] Lu Y Kiechl SJ Wang J Xu Q Kiechl S Pechlaner R. Global distributions of age- and sex-related arterial stiffness: systematic review and meta-analysis of 167 studies with 509,743 participants. EBioMedicine. (2023) 92:104619. 10.1016/j.ebiom.2023.104619 37229905 PMC10327869

[5] NCD Risk Factor Collaboration (NCD-RisC). Worldwide trends in underweight and obesity from 1990 to 2022: a pooled analysis of 3663 population-representative studies with 222 million children, adolescents, and adults. Lancet. (2024) 403:1027–50. 10.1016/S0140-6736(23)02750-2 38432237 PMC7615769

[6] Gomez-Zorita S Queralt M Vicente MA Gonzalez M Portillo MP. Metabolically healthy obesity and metabolically obese normal weight: a review. J Physiol Biochem. (2021) 77:175–89. 10.1007/s13105-020-00781-x 33704694

[7] Fan J Song Y Chen Y Hui R Zhang W. Combined effect of obesity and cardio-metabolic abnormality on the risk of cardiovascular disease: a meta-analysis of prospective cohort studies. Int J Cardiol. (2013) 168:4761–8. 10.1016/j.ijcard.2013.07.230 23972953

[8] Yeh TL Chen HH Tsai SY Lin CY Liu SJ Chien KL. The relationship between metabolically healthy obesity and the risk of cardiovascular disease: a systematic review and meta-analysis. J Clin Med. (2019) 8:1228. 10.3390/jcm8081228 31443279 PMC6723711

[9] Yoo HJ Hwang SY Hong HC Choi HY Seo JA Kim SG. Association of metabolically abnormal but normal weight (MANW) and metabolically healthy but obese (MHO) individuals with arterial stiffness and carotid atherosclerosis. Atherosclerosis. (2014) 234:218–23. 10.1016/j.atherosclerosis.2014.02.033 24681911

[10] Yuan Y Mu J-J Chu C Zheng W-L Wang Y Hu J-W. Effect of metabolically healthy obesity on the development of arterial stiffness: a prospective cohort study. Nutr Metab (Lond). (2020) 17:50. 10.1186/s12986-020-00474-8 32625239 PMC7330959

[11] Wang A Wang Y Zuo Y Tian X Chen S Ma Y. Risk of arterial stiffness according to metabolically healthy obese phenotype: a combined cross-sectional and longitudinal study in Kailuan cohort. Aging (Albany NY). (2021) 13:15114–25. 10.18632/aging.203075 34081619 PMC8221340

[12] Guo W Jia J Zhan M Li X Zhu W Lu J. Association of metabolically unhealthy non-obese and metabolically healthy obese individuals with arterial stiffness and 10-year cardiovascular disease risk: a cross-sectional study in Chinese adults. Nutr J. (2023) 22:44. 10.1186/s12937-023-00870-9 37726745 PMC10510138

[13] Townsend RR Wilkinson IB Schiffrin EL Avolio AP Chirinos JA Cockcroft JR. Recommendations for improving and standardizing vascular research on arterial stiffness: a scientific statement from the American Heart Association. Hypertension. (2015) 66:698–722. 10.1161/HYP.0000000000000033 26160955 PMC4587661

[14] Tomiyama H Shiina K. State of the art review: brachial-ankle PWV. J Atheroscler Thromb. (2020) 27:621–36. 10.5551/jat.RV17041 32448827 PMC7406407

[15] Park JB Sharman JE Li Y Munakata M Shirai K Chen CH. Expert consensus on the clinical use of pulse wave velocity in Asia. Pulse (Basel). (2022) 10:1–18. 10.1159/000528208 36660436 PMC9843646

[16] Bosco G Di Giacomo Barbagallo F Di Marco M Scilletta S Miano N Pistritto C. Translating the effect of dual lipid reduction with PCSK9 inhibitors on a mechanical vascular instrumental biomarker in familial hypercholesterolemia subjects. J Transl Med. (2025) 23:1371. 10.1186/s12967-025-07432-z 41331636 PMC12673767

[17] Ananda RA Solomon B Ray KK. Individual and joint associations of obesity and metabolic health parameters on arterial stiffness: evidence from the UK biobank. Diabetes Obes Metab. (2025) 27:899–910. 10.1111/dom.16090 39587367 PMC11701184

[18] Ding C Chan Z Magkos F. Lean, but not healthy: the “metabolically obese, normal-weight” phenotype. Curr Opin Clin Nutr Metab Care. (2016) 19:408–17. 10.1097/MCO.0000000000000317 27552473

[19] Aroor AR Jia G Sowers JR. Cellular mechanisms underlying obesity-induced arterial stiffness. Am J Physiol Regul Integr Comp Physiol. (2018) 314:R387–98. 10.1152/ajpregu.00235.2016 29167167 PMC5899249

[20] Hill MA Yang Y Zhang L Sun Z Jia G Parrish AR. Insulin resistance, cardiovascular stiffening and cardiovascular disease. Metab Clin Exp. (2021) 119:154766. 10.1016/j.metabol.2021.154766 33766485

[21] Kim H-L Lee J-M Seo J-B Chung W-Y Kim S-H Zo J-H. The effects of metabolic syndrome and its components on arterial stiffness in relation to gender. J Cardiol. (2015) 65:243–9. 10.1016/j.jjcc.2014.05.009 25034706

[22] Zuo J Chao H Tang B Avolio AP Schlaich MP Nolde JM. Female gender is associated with higher susceptibility of weight induced arterial stiffening and rise in blood pressure. J Clin Med. (2021) 10:3479. 10.3390/jcm10163479 34441775 PMC8396951

[23] Ogola BO Zimmerman MA Clark GL Abshire CM Gentry KM Miller KS. New insights into arterial stiffening: does sex matter? Am J Physiol Heart Circ Physiol. (2018) 315:H1073–87. 10.1152/ajpheart.00132.2018 30028199 PMC6415742

[24] Kehmeier MN Walker AE. Sex differences in large artery stiffness: implications for cerebrovascular dysfunction and Alzheimer's Disease. Front Aging. (2021) 2:791208. 10.3389/fragi.2021.791208 35072153 PMC8782423

