# Supplementary material for: Sex- and age-specific associations between metabolic phenotypes and brachial-ankle pulse wave velocity in adults: a cross-sectional study
Source: Front Med (Lausanne). 2026 Sep 25;13:1915757. doi: 10.3389/fmed.2026.1915757 (PMC13649661; doi:10.3389/fmed.2026.1915757)
Supplement: Supplementary file 1 [file Table1.docx]

**Supplementary Table S1. Firth penalized logistic regression sensitivity analysis for elevated baPWV**

| **Sex-age stratum** | **Phenotype** | **Primary Model 2 OR (95% CI)** | **Primary P** | **Firth OR (95% profile CI)** | **Firth PLR P** | **Firth FDR P** |
| --- | --- | --- | --- | --- | --- | --- |
| Men <50 | MHO | 1.32 (0.55-3.17) | 0.531 | 1.35 (0.55-3.11) | 0.493 | 0.493 |
| Men <50 | MONW | 7.68 (3.55-16.62) | <0.001 | 7.65 (3.54-16.39) | <0.001 | <0.001 |
| Men <50 | MUO | 4.61 (2.44-8.74) | <0.001 | 4.51 (2.45-8.71) | <0.001 | <0.001 |
| Men ≥50 | MHO | 0.85 (0.55-1.30) | 0.448 | 0.85 (0.55-1.30) | 0.469 | 0.493 |
| Men ≥50 | MONW | 3.37 (2.29-4.96) | <0.001 | 3.37 (2.28-4.93) | <0.001 | <0.001 |
| Men ≥50 | MUO | 2.34 (1.73-3.17) | <0.001 | 2.34 (1.73-3.16) | <0.001 | <0.001 |
| Women <50 | MHO | 2.94 (0.69-12.61) | 0.146 | 3.12 (0.71-11.98) | 0.123 | 0.148 |
| Women <50 | MONW | 6.12 (1.42-26.42) | 0.015 | 6.45 (1.46-24.93) | 0.017 | 0.025 |
| Women <50 | MUO | 6.60 (1.72-25.32) | 0.006 | 6.59 (1.72-23.91) | 0.008 | 0.015 |
| Women ≥50 | MHO | 0.92 (0.61-1.39) | 0.688 | 0.93 (0.61-1.39) | 0.713 | 0.713 |
| Women ≥50 | MONW | 2.58 (1.77-3.77) | <0.001 | 2.58 (1.77-3.76) | <0.001 | <0.001 |
| Women ≥50 | MUO | 2.36 (1.71-3.25) | <0.001 | 2.35 (1.71-3.24) | <0.001 | <0.001 |

*Firth model: phenotype (MHNW reference) + age (continuous) + current smoking + current alcohol consumption.*

*Inference: Firth bias-reduced penalized likelihood; 95% profile penalized-likelihood CI; penalized likelihood-ratio (PLR) P values.*

*FDR correction was applied within each sex across six phenotype comparisons.*

**Supplementary Table S2. Continuous baPWV sensitivity analysis using ln(baPWV)**

| **Sex-age stratum** | **Phenotype** | **β for ln(baPWV)** | **Adjusted % difference vs MHNW (95% CI)** | **P value** | **FDR P** |
| --- | --- | --- | --- | --- | --- |
| Men <50 | MHO | -0.00239 | -0.24% (-1.21% to 0.74%) | 0.632 | 0.632 |
| Men <50 | MONW | 0.06429 | 6.64% (4.69% to 8.63%) | <0.001 | <0.001 |
| Men <50 | MUO | 0.04293 | 4.39% (3.32% to 5.47%) | <0.001 | <0.001 |
| Men ≥50 | MHO | -0.00547 | -0.55% (-2.26% to 1.20%) | 0.537 | 0.632 |
| Men ≥50 | MONW | 0.09512 | 9.98% (7.40% to 12.62%) | <0.001 | <0.001 |
| Men ≥50 | MUO | 0.07119 | 7.38% (5.59% to 9.20%) | <0.001 | <0.001 |
| Women <50 | MHO | 0.01020 | 1.03% (-0.60% to 2.68%) | 0.219 | 0.241 |
| Women <50 | MONW | 0.07532 | 7.82% (5.24% to 10.47%) | <0.001 | <0.001 |
| Women <50 | MUO | 0.08048 | 8.38% (6.16% to 10.64%) | <0.001 | <0.001 |
| Women ≥50 | MHO | 0.01123 | 1.13% (-0.75% to 3.04%) | 0.241 | 0.241 |
| Women ≥50 | MONW | 0.09395 | 9.85% (7.46% to 12.29%) | <0.001 | <0.001 |
| Women ≥50 | MUO | 0.08689 | 9.08% (7.15% to 11.04%) | <0.001 | <0.001 |

*Outcome: natural-log-transformed mean bilateral baPWV. OLS models were adjusted for age, current smoking, and current alcohol consumption, with HC3 heteroskedasticity-robust standard errors.*

*β coefficients were exponentiated and expressed as adjusted percentage differences relative to MHNW.*

*FDR correction was applied within each sex across six phenotype comparisons.*
